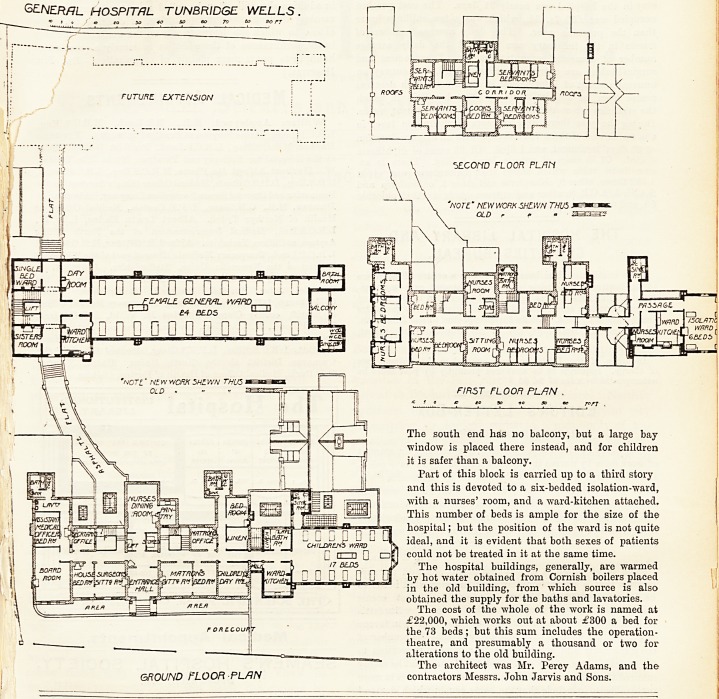# The New Hospital at Tunbridge Wells

**Published:** 1905-09-23

**Authors:** 


					THE NEW HOSPITAL AT TUNBRIDGE WELLS.
Like so many buildings of its age and class, this hospital
Soccame completely out of date, and it was wisely decided by
the governors to build new wards for the patients, and to
utilise the old hospital as an administrative department. The
new out-patients' department is placed at the south-west
?corner of the building, and it comprises a large waiting-hall
?with its separate entrance, and 'adjoining it are the various
?consulting-rooms and examining-rooms, dispensary, medicine
waiting-hall, and a separate exit, so that these out-patients
?enter at one door, pass through the waiting-hall, consulting-
room and examining-room, and so outwards without retracing
their steps.
The old main entrance to the hospital lias been retained,
and from the entrance-hall down a flight of steps the lower
ground-floor is reached. From the waiting-hall of the
?casualty-room springs a curved corridor about 30 feet long
which gives access to the new wards for male patients. At
the entrance to this new ward is the sisters' room on the left
hand ; then the staircase, with a large lift incorporated, and
the single-bedded ward. Opposite the lift is the entrance to
the men's ward,' having on one side the ward-kitchen and
on the other a small day-room. The ward has its long
ficcess nearly north and south, which is a very good aspect.
The ward is about 100 feet long and 27 feet wide, giving
rather more than 112 superficial feet per bed, and a trifle
more than 8 feet of wall space per bed. It can hardly be
said that either of these is excessive, and wfe should say that
the latter is insufficient for a modern hospital; but, of
course, much more will depend upon the amount of fresh air
supplied than on the mere superficial or cubic space, and it
has to be commended that in this ward every bed has a
window on both sides. The single-bedded ward has a window
on its east and north walls, and so obtains a considerable
amount of cross-current; but it would have been better
to project the room another 4 feet to the north, and
so obtain direct cross - ventilation from east to west.
The sanitary annexes are placed at the southern end of
the ward, and they are well cut oif from the ward by double
doors and cross-ventilated passages. Between these annexes
there is a good balcony which, facing nearly south, must
prove a most useful adjunct to the ward.
It is evident that much care has been expended on the
building. The corners of the ward walls and the usual
ledges have been rounded off or omitted to prevent the
accumulation of dust and to render cleaning easier, and all
pipes have been fixed a few inches away from the walls. The
456 THE HOSPITAL. Sept.|23, 1905.
wards have a 5-feet dado of pale-blue tiles, and above this
dado the walls are painted white. The floors of the wards
are of concrete and covered with linoleum. It is well known
that linoleum forms an admirable floor covering for wards,
and if the joinings can be made perfectly close when laid on
concrete, the plan is one which ought to find favour in
hospitals, as it will be found warmer and more economical
than terrazzo and, above all, it will not crack. The wards are
warmed by stoves placed in the centre of the floor. Flues for the
entrance of fresh air and for the exit of the smoke run under the
floors. So far as the plans sent to us show, there are no hot-water
radiators placed in the wards, but their omission is one we
do not much regret, for although this " artificial" system of
warming the wards may be useful occasionally, its habitual
use is almost sure to be injurious, and we are therefore in-
clined to think that it is safer to trust to open fires, as is done-
at some of our more modern hospitals.
Following the corridor through the end of the block we
reach the operating - theatre, with its waiting - room and
anaesthetic-room. The walls of these rooms are covered with
glazed tiles, and the floors are laid with terrazzo. The
theatre is properly lighted with a large north window, and
radiators are supplied, so that the temperature can be quickly
raised when necessary.
The ground-floor, as far as the ward goes, is exactly the
same as the lower ground-floor, and it is reached by the same
curved corridor. Over the out-patients' department is the
children's ward for 17 beds. This ward is quite well arranged.
GENERAL HOSPITAL.. TUNBRIDGE WELLS
/o 3 o *> .? to +o so eo jo & sc pT
Sept. 23, 1905. THE HOSPITAL. 457
The south end has no balcony, but a large bay
window is placed there instead, and for children
it is safer than a balcony.
Part of this block is carried up to a third story
and this is devoted to a six-bedded isolation-ward,
with a nurses' room, and a ward-kitchen attached.
This number of beds is ample for the size of the
hospital; but the position of the ward is not quite
ideal, and it is evident that both sexes of patients
could not be treated in it at the same time.
The hospital buildings, generally, are warmed
by hot water obtained from Cornish boilers placed
in the old building, from which source is also
obtained the supply for the baths and lavatories.
The cost of the whole of the work is named at
?22,000, which works out at about ?300 a bed for
the 73 beds; but this sum includes the operation-
theatre, and presumably a thousand or two for
alterations to the old building.
The architect was Mr. Percy Adams, and the
contractors Messrs. John Jarvis and Sons.
GENERAL hospital tunbridge wells.
o o to yo 40 so go 70 to so rT
\
FUTURE EXTENSION
The south end has no balcony, but a large bay
window is placed there instead, and for children
it is safer than a balcony.
Part of this block is carried up to a third story
and this is devoted to a six-bedded isolation-ward,
with a nurses' room, and a ward-kitchen attached.
This number of beds is ample for the size of the
hospital; but the position of the ward is not quite
ideal, and it is evident that both sexes of patients
could not be treated in it at the same time.
The hospital buildings, generally, are warmed
by hot water obtained from Cornish boilers placed
in the old building, from which source is also
obtained the supply for the baths and lavatories.
The cost of the whole of the work is named at
?22,000, which works out at about ?300 a bed for
the 73 beds; but this sum includes the operation-
theatre, and presumably a thousand or two for
alterations to the old building.
The architect was Mr. Percy Adams, and the
GROUND FLOOR PLAN contractors Messrs. John Jarvis and Sons.

				

## Figures and Tables

**Figure f1:**
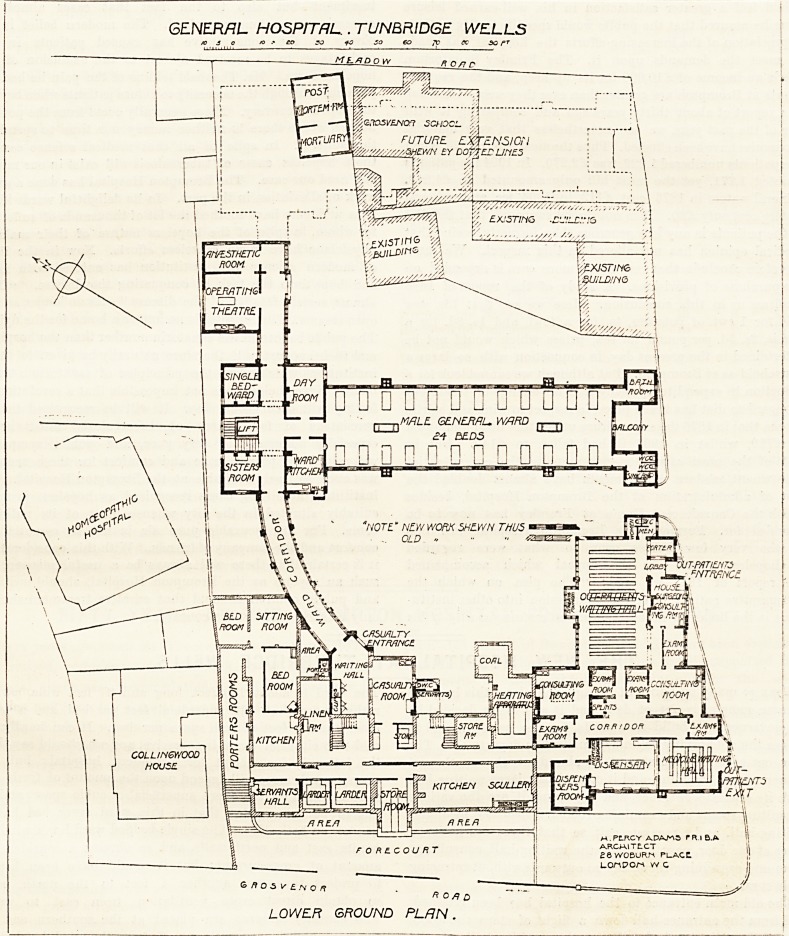


**Figure f2:**